# Pancreatic Cancer Incidence Trends by Race, Ethnicity, Age and Sex in the United States: A Population-Based Study, 2000–2018

**DOI:** 10.3390/cancers15030870

**Published:** 2023-01-31

**Authors:** Jamil S. Samaan, Yazan Abboud, Janice Oh, Yi Jiang, Rabindra Watson, Kenneth Park, Quin Liu, Katelyn Atkins, Andrew Hendifar, Jun Gong, Arsen Osipov, Debiao Li, Nicholas N. Nissen, Stephen J. Pandol, Simon K. Lo, Srinivas Gaddam

**Affiliations:** 1 Karsh Division of Gastroenterology and Hepatology, Cedars-Sinai Medical Center, 8700 Beverly Blvd, Suite 7705, Los Angeles, CA 90048, USA; 2 Department of Radiation Oncology, Cedars-Sinai Medical Center, Los Angeles, CA 90048, USA; 3 Department of Medicine, Samuel Oschin Comprehensive Cancer Institute, Cedars-Sinai Medical Center, Los Angeles, CA 90048, USA; 4 Biomedical Imaging Research Institute, Cedars-Sinai Medical Center, Los Angeles, CA 90048, USA; 5 Department of Hepatobiliary and Pancreatic Surgery, Cedars-Sinai Medical Center, Los Angeles, CA 90048, USA

**Keywords:** cancer disparity, sex disparity, gender, pancreatic cancer trends, ethnicity, race/racial, white/caucasian, black/african american, hispanic/latinx, disparity/disparities

## Abstract

**Simple Summary:**

Pancreatic cancer (PC) incidence is increasing at a greater rate in young women compared to young men. We aimed to understand the association of race and ethnicity with these trends by performing race, ethnicity and age-specific analysis using the SEER 21 database. We organized race and ethnicity groups by Non-Hispanic White (White), Non-Hispanic Black (Black) and Hispanic, and age groups as older adults (age ≥ 55 years) and younger adults (age < 55 years). We found a greater rate of increase in PC incidence among young women compared to young men among all race and ethnicity groups, although young Hispanic and Black women experienced a disproportionately greater increase. When comparing trends among women from all race and ethnicity groups, young Hispanic women experienced a greater rate of increase in PC incidence compared to young White and Black women. Our study demonstrates the association of race and ethnicity with PC incidence trends and highlights the disproportionate burden of disease on young women of color.

**Abstract:**

Background and aims: Pancreatic cancer (PC) incidence is increasing at a greater rate in young women compared to young men. We performed a race- and ethnicity-specific evaluation of incidence trends in subgroups stratified by age and sex to investigate the association of race and ethnicity with these trends. Methods: Age-adjusted PC incidence rates (IR) from the years 2000 to 2018 were obtained from the SEER 21 database. Non-Hispanic White (White), Non-Hispanic Black (Black) and Hispanic patients were included. Age categories included older (ages ≥ 55) and younger (ages < 55) adults. Time-trends were described as annual percentage change (APC) and average APC (AAPC). Results: Younger White [AAPC difference = 0.73, *p* = 0.01)], Black [AAPC difference = 1.96, *p* = 0.01)] and Hispanic [AAPC difference = 1.55, *p* = 0.011)] women experienced a greater rate of increase in IR compared to their counterpart men. Younger Hispanic women experienced a greater rate of increase in IR compared to younger Black women [AAPC difference = −1.28, *p* = 0.028)] and younger White women [AAPC difference = −1.35, *p* = 0.011)]. Conclusion: Younger women of all races and ethnicities experienced a greater rate of increase in PC IR compared to their counterpart men; however, younger Hispanic and Black women experienced a disproportionately greater increase. Hispanic women experienced a greater rate of increase in IR compared to younger Black and White women.

## 1. Introduction

Despite substantial diagnostic and therapeutic medical advancements in recent decades, pancreatic cancer (PC) remains a deadly disease with an estimated 5-year survival rate of 10.8% [1]. Patients are often diagnosed after regional or distant metastasis, with only 11.3% diagnosed at an early stage [1]. Patients with PC accrue more than 15 times the healthcare costs of patients without PC [2]. Furthermore, these patients suffer decreases in quality in all domains of life and worse psychological quality of life when compared to patients with other cancers [3]. In the year 2021, PC will be the 11th and 3rd overall leading cause of cancer incidence and mortality in the United States (US), respectively [1].

Our recent study examining national time trends in PC incidence using the Surveillance, Epidemiology and End Result (SEER) database revealed sex disparity in PC incidence trends over the past two decades [4]. When stratified by age and sex, younger women (ages < 55) were found to have a significantly higher relative rate of increase in PC incidence compared to younger men. Interestingly, this sex disparity trend was not observed in the older population (ages ≥ 55), posing the possibility of a distinct driver(s) in incidence that may be unique to younger women. However, this study does not give information on whether racial differences accounted for the sharp rise in incidence in younger women.

Racial disparities in the incidence and outcomes of multiple gastrointestinal malignancies have been previously demonstrated, including higher PC incidence and mortality rates among Black compared to White persons [5,6]. Therefore, the aim of the current study was to perform race- and ethnicity-specific evaluations of incidence trends in subgroups stratified by age and sex using a large population-based SEER database.

## 2. Materials and Methods

### 2.1. Data Source

The SEER program is a cancer incidence and survival database compiled from population-based cancer registries across the US and provided by the National Cancer Institute (NCI). We used the SEER 21, which covers 36.4% of the US population and reports cancer cases from the year 2000 to 2018 [7,8]. The SEER 21 database is the most comprehensive compared to the remaining available databases, providing data from more geographical regions and including a larger proportion of the US population. Further, this database provides information on reporting delays. Given this data is publicly available and is de-identified, according to Cedars-Sinai institutional policy, this study was considered exempt from the institutional review board’s (IRB) full or expedited review protocol. This is in accordance with the recommendations of the National Human Research Protections Advisory Committee.

### 2.2. Definitions

Incidence rates are reported as the number of cases per 100,000 population. The percent change in PC incidence between two consecutive years was defined as the annual percent change (APC), which describes magnitude as well as directionality. The average APC (AAPC) was calculated using the overall change in incidence divided by the total number of years in the study period. We organized race and ethnicity groups into the following: Non-Hispanic White (White), Non-Hispanic Black (Black) and Hispanic, which includes White Hispanic and Black Hispanic patients [9]. Due to the small sample size, the patient groups Asian/Pacific Islander, American Indian/Alaskan Native and Native Hawaiian were not included in our analysis. Age stratification was performed by using an age cut-off of 55 years: Older adults (age ≥ 55 years) and younger adults (age < 55 years). This cutoff was determined based on precedent from previous publications as well as the examination of the distribution of cases by age groups as shown in Figure S1 [4,10].

### 2.3. Data Retrieval and Study Period

PC incidence data from 1 January 2000–31 December 2018 were retrieved using the software SEER*Stat (NCI), version 8.3.9.2. PC cases were identified using the International Classification of Diseases for Oncology, third edition, “Site Recode ICD-O-3/WHO 2008 classification”.

### 2.4. Statistical Analysis

The number of cancer cases is presented as frequencies and percentages, while incidence rates are presented as cases per 100,000 population. The software SEER*Stat (NCI), version 8.3.9.2, was used to calculate the annual PC incidence. Incidence rates were age-adjusted for the 2000 US population and for reporting delays. Joinpoint Regression Program v4.9.0.0 was used to generate best-fit models on a logarithmic scale [11]. Parametric estimations were used to calculate APC and AAPC for the study period [12,13]. The statistical difference from zero was assessed using a two-sided *t*-test for the APC and AAPC, while the tests of parallelism and coincidence were used to assess if trends were parallel or identical, respectively, between the AAPCs of segmented-linear trends [14]. The test of parallelism determines if two trends are parallel to each other by evaluating if the trends have a statistically significant difference in slope. The test of coincidence, sometimes referred to as the test of identicalness, assesses if the rates of one trend are identical or statistically different from the rates of the other trend. The two *p*-values generated from these two tests are comparative in nature between trends in subgroups. The concepts of parallelism and coincidence are illustrated in Figure S2.

The test of parallelism was conducted using log-linear models on the log-transformed scale of the APCs. Subsequently, the Joinpoint Regression Program back-transforms the results to the original scale and provides a report [14,15]. To estimate the statistical significance of the difference between AAPCs, Taylor Series expansion was used [16]. These methods were used to examine sex disparity in PC incidence trends when stratified by age, race and ethnicity, and further analysis examined race and ethnic disparity in cancer incidence among women stratified by age, race and ethnicity. A 2-sided *p*-value of less than 0.05 was considered statistically significant.

## 3. Results

### 3.1. Demographics

A total of 283,817 PC cases were reported during the study period. The majority occurred in women (50.1%) and White patients (72.7%). A total of 88.6% of patients were diagnosed at an age greater than or equal to 55, with a median age of 71 years old. The SEER 21 database reports cancer data on 36.4% of the US population, including 33.6%, 44.7%, and 46.7% of the White, Black and Hispanic populations in the US, respectively [8]. A summary of PC incidence rates stratified by age, sex, race and ethnicity is shown in Table 1.

### 3.2. Older Adults (Age ≥ 55)

A total of 251,360 cases of PC were reported during the study period. Sex-specific incidence rates per 100,000 were 46.49 (95% CI: 46.23–46.75) in women and 58.97 (95% CI: 58.63–59.30) in men.

**Figure 1:**
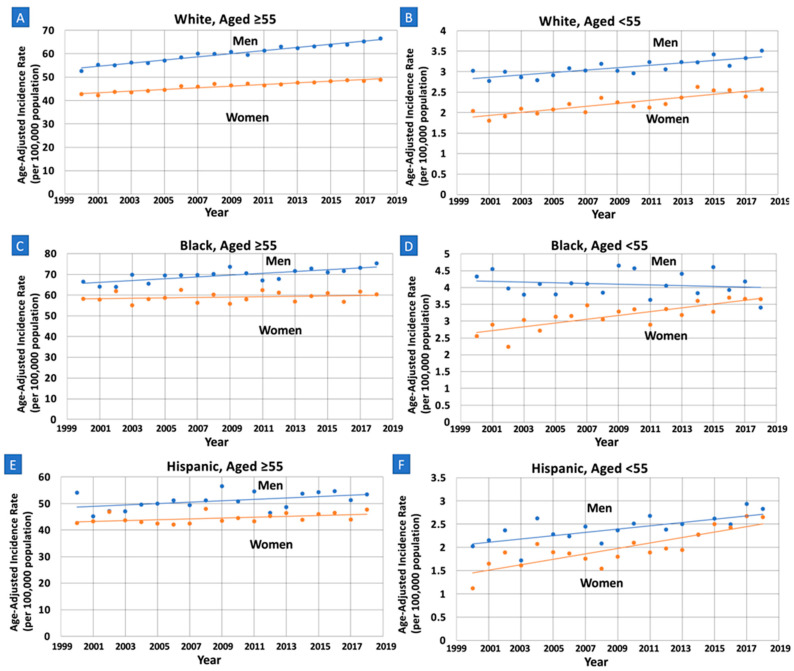
Sex-specific trends and age-adjusted pancreatic cancer incidence rates per 100,000 people among older (Age ≥ 55) and younger (Age < 55) White, Black and Hispanic adults. APC: annual percent change. (**A**): Older White: APC increasing at a lower rate in women compared to men (0.80 vs. 1.12, *p* = 0.001), with trends that are non-parallel (*p* < 0.01) and non-identical (*p* < 0.01). (**B**): Younger White: APC increasing at a greater rate in women compared to men (1.68 vs. 0.94, *p* = 0.010), with trends that are non-parallel (*p* < 0.01) and non-identical (*p* < 0.01). (**C**): Older Black: APC increasing at a lower rate in women compared to men (0.17 vs. 0.63, *p* = 0.026), with trends that are parallel (*p* = 0.071) and non-identical (*p* < 0.01). (**D**): Younger Black: APC increasing at a greater rate in women compared to men (1.74 vs. −0.22, *p* < 0.001), with trends that are non-parallel (*p* < 0.01) and non-identical (*p* < 0.01). (**E**): Older Hispanic: APC neither increasing or decreasing in women and men, no significant difference (0.38 vs. 0.51, *p* = 0.65). (**F**): Younger Hispanic: APC increasing at a greater rate in women compared to men (3.03 vs. 1.48, *p* = 0.011); trends are non-parallel (*p* < 0.01) and non-identical (*p* < 0.01).

Incidence rates were relatively increasing at a lower rate in White women (AAPC = 0.80, 95% CI: 0.64–0.96, *p* < 0.01) compared to White men (AAPC = 1.12, 95% CI: 1.00–1.24, *p* < 0.01), with a statistically significant AAPC difference of −0.32 (95% CI: −0.52–−0.13, *p* < 0.01) (Table 2, Figure 1). These trends were neither identical (*p* < 0.01) nor parallel (*p* < 0.01). No sex differences in incidence rates were found in the Black and Hispanic groups.

**Figure 2:**
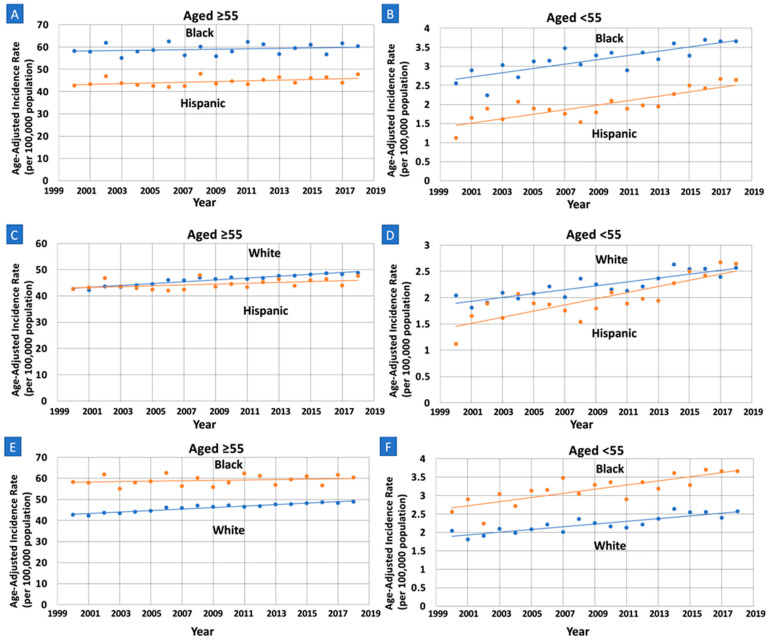
Race and ethnicity specific trends and age-adjusted pancreatic cancer incidence rates per 100,000 people among older (Age ≥ 55) and younger women (Age < 55). APC: annual percent change. (**A**): APC increasing in Hispanics and unchanged in Black older women; there is no significant difference (0.38 vs. 0.17, *p* = 0.34). (**B**): APC increasing at a greater rate in Hispanic compared to Black younger women (3.03 vs. 1.74, *p* = 0.028), with trends that are parallel (*p* = 0.074) and non-identical (*p* < 0.01). (**C**): APC increasing at a greater rate in White compared to Hispanic older women (0.80 vs. 0.38, *p* = 0.021), with trends that are non-parallel (*p* = 0.016) and non-identical (*p* < 0.01). (**D**): APC increasing at a greater rate in Hispanic compared to White younger women (3.03 vs. 1.68, *p* < 0.001), with trends that are parallel (*p* = 0.070) and non-identical (*p* < 0.01). (**E**): APC increasing at a greater rate in White compared to Black older women (0.80 vs. 0.17, *p* = 0.001), with trends that are non-parallel (*p* < 0.01) and non-identical (*p* < 0.01). (**F**): APC increasing in Black and White younger women, with no significant difference (1.74 vs. 1.68, *p* = 0.87).

Incidence rates were also relatively higher in White women (AAPC = 0.80, 95% CI: 0.64–0.96, *p* < 0.01) compared to Hispanic women (AAPC = 0.38, 95% CI: 0.05–0.72, *p* = 0.027), with a difference in AAPC of 0.41 (95% CI: 0.06–0.76, *p* = 0.021) (Table 3, Figure 2). These trends were also neither identical (*p* < 0.01) or parallel (*p* = 0.016). Furthermore, incidence rates were relatively higher in White women (AAPC = 0.80, 95% CI: 0.64–0.96, *p* < 0.01) compared to Black women (AAPC = 0.17, 95% CI: −0.18–0.51, *p* = 0.32), with a difference in AAPC of 0.63 (95% CI: 0.27–0.99, *p* < 0.01). These trends were neither identical (*p* < 0.01) nor parallel (*p* < 0.01).

## 4. Younger Adults (Ages < 55)

A total of 32,369 cases of PC were reported during the study period. Most cases occurred among men (6.5% of all cases), and the sex-specific incidence rates per 100,000 were 2.25 (95% CI: 2.21–2.28) in women and 3.01 (95% CI: 2.96–3.05) in men.

Incidence rates were relatively increasing at a greater rate in women compared to men in all three race and ethnic groups. White women (AAPC = 1.68, 95% CI: 1.18–2.17, *p* < 0.01) experienced a greater rate of increase in incidence rates compared to White men (AAPC = 0.94, 95% CI: 0.61–1.28, *p* < 0.01) with a statistically significant AAPC difference of 0.73 (95% CI: 0.18–1.29, *p* = 0.01). These trends were neither identical (*p* < 0.01) nor parallel (*p* < 0.01). Black women (AAPC = 1.74, 95% CI: 1.04–2.46, *p* < 0.01) experienced an increase in incidence rate compared to Black men (AAPC = −0.22, 95% CI: −1.01–0.48, *p* = 0.57), with a statistically significant AAPC difference of 1.96 (95% CI: 0.98–2.95, *p* < 0.01). These trends were neither identical (*p* < 0.01) nor parallel (*p* = 0.02). Hispanic women (AAPC = 3.03, 95% CI: 2.03–4.04, *p* < 0.01) experienced a greater rate of increase in incidence rates compared to Hispanic men (AAPC = 1.48, 95% CI: 0.68–2.82, *p* < 0.01), with a statistically significant AAPC difference of 1.55 (95% CI: 0.36–2.75, *p* = 0.011). These trends were neither identical (*p* < 0.01) nor parallel (*p* < 0.01).

Incidence rates were relatively higher in Hispanic women (AAPC = 3.03, 95% CI: 2.03–4.04, *p* < 0.01) compared to Black women (AAPC = 1.74, 95% CI: 1.04–2.46, *p* < 0.01), with a difference in AAPC of −1.28 (95% CI: −2.42–−0.14, *p* = 0.03). These trends were non-identical (*p* < 0.01) and parallel (*p* = 0.07). Furthermore, incidence rates were relatively higher in Hispanic women (AAPC = 3.03, 95% CI: 2.03–4.04, *p* < 0.01) compared to White women (AAPC = 1.68, 95% CI: 1.18–2.17, *p* < 0.01), with a difference in AAPC of −1.35 (95% CI: − 2.39–−0.31, *p* = 0.01). These trends were non-identical (*p* < 0.01) and parallel (*p* = 0.07).

## 5. Discussion

We investigated our recent finding of an alarming increase in PC incidence rates in young women compared to young men by examining the association of race and ethnicity with these trends [4]. Initially, we examined sex disparity in PC incidence time trends stratified by age, race and ethnicity. While younger women (age < 55) showed a greater relative rate of increase in PC incidence compared to young men in all races and ethnic groups, rates disproportionately increased in younger Hispanic and Black women. Further analysis revealed that younger Hispanic women experienced significantly higher rates of increase in incidence compared to younger Black and White women. The present analysis builds on our previous findings by identifying racial and ethnic disparities in PC incidence, specifically in younger women. Given PC is expected to become the second leading cause of cancer death by the year 2030 in the US [17], we hope our findings aid in designing future studies and public health strategies to combat these alarming trends.

A racial gap in PC incidence has existed in the US since the 1970s with a greater incidence in Black compared to White patients, although known risk factors such as socioeconomic status (SES), lifestyle and biological variables do not alone independently explain these trends [18]. While our analysis did not investigate causative factors, the trends found in our analysis are likely due to changes in trends of modifiable as well as non-modifiable risk factors. SES has been shown to be associated with cancer incidence and outcomes, with the directionality of the relationship varying by cancer type [19]. A systematic review of European studies found variable effects of SES on PC incidence, varying by country of origin and, interestingly, patient sex [20]. Diabetes mellitus (DM), obesity and smoking have also been associated with PC incidence [21].

Obesity and DM have both been shown to disproportionately affect minority communities, although their impact on racial differences in PC incidence remains unclear [22,23]. An analysis of the NHANES surveys examined trends in the prevalence of diagnosed and undiagnosed DM in the United States from 1988 to 2012 and showed an increase in DM rates across all demographics, although interestingly only among those with a BMI of 30 or greater [24]. Furthermore, rates of undiagnosed DM only increased among Mexican American participants, with undiagnosed DM rates decreasing among all demographics except for Mexican Americans and the youngest age group, highlighting a possible disparity in access to care. A more recent study that examined trends in DM rates from 1999 to 2016 using the NHANES showed a significantly more dramatic increase in DM rates among Mexican Americans during the study period compared to the Non-Hispanic Black and Non-Hispanic White groups [25]. Interestingly, men experienced a greater increase in DM rates overall compared to women. Obesity trends have also been explored through the NHANES, with recent studies finding an overall increase in obesity rates over the past few decades, with higher rates in younger cohorts compared to older [26,27]. While similar trend patterns of severe obesity were found on subgroup analysis by demographics, males were less likely to have severe obesity compared to females in all birth cohorts [27]. Lastly, a recent review shows smoking rates have decreased among all racial and ethnic groups in both men and women in recent decades, with Asian and Hispanic/Latino individuals demonstrating the lowest prevalence [28]. The review also highlights significant racial, ethnic and socioeconomic disparities in exposure to secondhand smoke, which may further drive disparities in risk. The literature is limited by a lack of simultaneous stratification of risk factors by race, ethnicity, sex and age. Furthermore, the relationship between the trends found in our analysis and these risk factors, along with other known and unknown risk factors, is complex and warrants further investigation.

The association of sex-specific factors with PC has also been investigated. A meta-analysis of 22 studies found higher parity was borderline 0.86 (95% CI: 0.73–1.02; *Q* = 50.49, *p* < 0.001, *I*^2^ = 58.4%) associated with a dose response decreased risk of PC when controlling for possible confounders such as smoking and diabetes mellitus rates [29]. It is proposed that the protective effect of pregnancy is likely sex hormone mediated. Estrogen has been shown in in vitro and in vivo studies to inhibit transplanted pancreatic carcinoma and the effects of early pancreatic carcinogenesis in rat models [30,31]. Furthermore, census data shows a decline in the overall birth rate in the US from 1980 to 2007 [32]. When stratified by race, birth rates significantly declined among Black women (84.9 to 72.7 births/1000 women) while birth rates mildly increased in White women (65.6 to 68.8 births/1000 women), although comparative analysis was not conducted between the races [32]. Unfortunately, birth rates for the Hispanic population were not available in this report.

The average age of diagnosis of PC is 71 years old, with up to 8% of patients diagnosed before the age of 50 [1,33]. Early-onset PC is relatively understudied, with an increasing incidence in recent years [33,34]. A recent analysis of 124,442 patients examined early and later age-onset PC incidence rates and found a higher percentage of men (58.3% vs. 49.8%), Black (16.8 vs. 12.2) and Hispanic (8.3 vs. 4.9) patients in the early group. Another study of 16,282 cases of PC also showed higher rates of Black (46%) and Hispanic (42%) patients diagnosed under the age of 65 compared to White (33%) and Asian (32%) patients (*p* < 0.0001) [35]. While early-onset PC has similar risk factors to late-onset PC, familial and genetic factors have been implicated as additional risk factors given that early-onset cases are more likely to have a family history of cancer and hereditary genetic syndromes [36]. Genetic factors are less likely to have had an impact on the change in trends revealed by our analysis due to the low likelihood of significant changes in the prevalence of genetic syndromes and hereditary diseases during our study period. Alternatively, we believe the disparity in incidence trends is likely due to changes in risk factor trends, both known and potentially unknown.

### Limitations and Future Directions

The SEER database has certain limitations, including possible loss of records and coding reliability. Underreporting of cases as well as changes in testing rates may impact incidence rates across our study period. Furthermore, demographic data, including race and ethnicity, are often obtained from multiple sources, such as patient intake, provider notes, and administrative databases, allowing for misclassification bias and an impact on our findings. Given the self-reported nature of race and ethnicity, this may not correspond to genetic ancestry, which is an important consideration when drawing conclusions from our analysis. Migration of patients in and out of SEER registry areas and selection bias are also possible limitations that have been previously described [37]. Furthermore, due to the small sample size, the patient groups Asian/Pacific Islander, American Indian/Alaskan Native and Native Hawaiian were not included in our analysis. Our analysis is also limited by the lack of availabile data on risk factors that may explain these trends, making definitive conclusions regarding causal factors not possible. The relatively low 5-year survival rate of PC makes prevention through risk modification and effective screening guidelines crucial. Examining national trends in cancer incidence, such as in our study, highlights alarming trends warranting further investigation, ultimately leading to public health policy actions. We highly encourage future studies examining factors associated with the alarming disproportionate increase in incidence rates in young Black and Hispanic women compared to their male counterparts, as well as the disproportionate increase in younger Hispanic women compared to younger Black and White women. Given that race is a social construct, examination of trends stratified by race and ethnicity provides important insight into the impact of psychosocial, societal, and systemic factors on PC incidence and outcomes. To further improve screening and therapeutics, we encourage future investigation of genomic profiles as objective tools for improving screening, prevention, and therapeutics given the expected rise in incidence in the coming decade.

## 6. Conclusions

Younger women of all races and ethnicities experienced a greater rate of increase in pancreatic cancer incidence compared to their male counterparts; however, younger Hispanic and Black women experienced a disproportionately greater increase. Comparison between younger women revealed that younger Hispanic women experienced significantly higher rates of increase in incidence compared to younger Black and White women. Further studies are needed to better understand the factors associated with these disparities.

## Figures and Tables

**Table 1:** Summary of age-adjusted pancreatic cancer incidence rates per 100,000 people stratified by age, sex, race and ethnicity.

	All Ages	Age ≥ 55	Age < 55
All Races and Ethnicities			
Women			
Cases (%)	142,297 (50.1)	128,204 (45.2)	14,035 (4.9)
Incidence Rate (95% CI)	11.22 (11.16, 11.28)	46.49 (46.23, 46.75)	2.25 (2.21, 2.28)
Men			
Cases (%)	141,520 (49.9)	123,156 (43.4)	18,334 (6.5)
Incidence Rate (95% CI)	14.32 (14.24, 14.39)	58.97 (58.63, 59.30)	3.01 (2.96, 3.05)
White			
Women			
Cases (%)	101,670 (49.3)	93,247 (45.2)	8400 (4.1)
Incidence Rate (95% CI)	11.11 (11.04, 11.18)	46.06 (45.76, 46.36)	3.08 (3.02, 3.14)
Men			
Cases (%)	104,731 (50.7)	92,927 (45.0)	11,792 (5.7)
Incidence Rate (95% CI)	14.6 (14.51, 14.69)	60.11 (59.72, 60.50)	2.21 (2.16, 2.26)
Black			
Women			
Cases (%)	17,579 (53.6)	15,159 (46.2)	2412 (7.4)
Incidence Rate (95% CI)	14.41 (14.19, 14.62)	58.93 (57.99, 59.89)	3.17 (3.04, 3.30)
Men			
Cases (%)	15,216 (46.4)	12,482 (38.1)	2732 (8.3)
Incidence Rate (95% CI)	17.25 (16.96, 17.55)	69.83 (68.53, 71.14)	4.08 (3.93, 4.24)
Hispanic			
Women			
Cases (%)	13,634 (51.9)	11,501 (43.8)	2114 (8.0)
Incidence Rate (95% CI)	10.65 (10.47, 10.84)	44.45 (43.63, 45.29)	2.01 (1.93, 2.10)
Men			
Cases (%)	12,647 (48.1)	10,162 (38.7)	2476 (9.4)
Incidence Rate (95% CI)	12.31 (12.08, 12.54)	51.13 (50.08, 52.19)	2.42 (2.32, 2.51)

Case percentages are reported as a percent of all cases; CI: confidence interval.

**Table 2:** Pancreatic cancer incidence time trends for White, Black and Hispanic patients from 2000 to 2018 stratified by age and sex.

	White	*p*	Black	*p*	Hispanic	*p*
All Ages						
Men AAPC (95% CI)	1.10 (0.99, 1.21)	<0.001	0.50 (0.25, 0.76)	<0.001	0.63 (0.18, 1.09)	0.009
Women ^‡^ AAPC (95% CI)	0.87 (0.74, 1.01)	<0.001	0.37 (0.10, 0.64)	0.011	0.68 (0.38, 0.99)	<0.001
ΔAAPC (95% CI)	−0.23 * (−0.39, −0.07)	0.005	−0.13 (−0.48, 0.22)	0.460	0.05 (−0.46, 0.56)	0.847
Age ≥ 55						
Men AAPC (95% CI)	1.12 (1.00, 1.24)	<0.001	0.63 (0.36, 0.89)	<0.001	0.51 (0.01, 1.02)	0.046
Women ^‡^ AAPC (95% CI)	0.80 (0.64, 0.96)	<0.001	0.17 (−0.18, 0.51)	0.322	0.38 (0.05, 0.72)	0.027
ΔAAPC (95% CI)	−0.32 * (−0.52, −0.13)	0.001	−0.46 (−0.86, −0.06)	0.026	−0.13 (−0.69, 0.43)	0.648
Age < 55						
Men AAPC (95% CI)	0.94 (0.61, 1.28)	<0.001	−0.22 (−1.01, 0.58)	0.569	1.48 (0.68, 2.82)	0.001
Women ^‡^ AAPC (95% CI)	1.68 (1.18, 2.17)	<0.001	1.74 (1.04, 2.46)	<0.001	3.03 (2.03, 4.04)	<0.001
ΔAAPC (95% CI)	0.73 * (0.18, 1.29)	0.010	1.96 * (0.98, 2.95)	<0.001	1.55 * (0.36, 2.75)	0.011

* AAPC trends used to calculate ΔAAPC are non-parallel and non-identical. ^‡^ Reference group when calculating ΔAAPC. Incidence trends were calculated per 100,000 people. AAPC: average annual percent change. CI: Confidence. Interval. ΔAAPC: difference in AAPC between women and men.

**Table 3:** Pancreatic cancer incidence time trends for women from 2000 to 2018 stratified by age, race and ethnicity as well as comparison of time trends between White, Black and Hispanic Women.

	All Ages	*p*	Age ≥ 55	*p*	Age < 55	*p*
White AAPC (95% CI)	0.87 (0.74, 1.01)	<0.001	0.80 (0.64, 0.96)	<0.001	1.68 (1.18, 2.17)	<0.001
Black AAPC (95% CI)	0.37 (0.10, 0.64)	0.011	0.17 (−0.18, 0.51)	0.322	1.74 (1.04, 2.46)	<0.001
Hispanic AAPC (95% CI)	0.68 (0.38, 0.99)	<0.001	0.38 (0.05, 0.72)	0.027	3.03 (2.03, 4.04)	<0.001
Trend Comparison Among Race and Ethnicity Cohorts						
Black ^‡^ vs. Hispanic ΔAAPC (95% CI)	−0.31 (−0.69, 0.07)	0.108	−0.22 (−0.66, 0.23)	0.343	−1.28 (−2.42, −0.14)	0.028
White ^‡^ vs. Black ΔAAPC (95% CI)	0.50 * (0.22, 0.79)	<0.001	0.63 * (0.27, 0.99)	0.001	−0.07 (−0.87, 0.73)	0.866
White ^‡^ vs. Hispanic ΔAAPC (95% CI)	0.19 (−0.12, 0.50)	0.222	0.41 * (0.06, 0.76)	0.021	−1.35 (−2.39, −0.31)	0.011

* AAPC trends used to calculate differences in AAPC are non-parallel and non-identical. **^‡^** Reference group when calculating ΔAAPC. Incidence trends were calculated per 100,000 people. AAPC: average annual percent change. CI: confidence interval. ΔAAPC: difference in AAPC between women and men.

## Data Availability

The data presented in this study are openly available at https://seer.cancer.gov/data-software/ (accessed on 22 January 2022).

## Supplementary Materials

The following supporting information can be downloaded at: https://www.mdpi.com/article/10.3390/cancers15030870/s1, Figure S1: Number of Pancreatic Cancer Cases During the Study Period (2000–2018) by Age Groups. Figure S2: Visual illustration of the test of Parallelism (Parallel Lines) and test of Coincidence (Identical Lines).
