# Supplementary material for: Pancreatic Cancer Incidence Trends by Race, Ethnicity, Age and Sex in the United States: A Population-Based Study, 2000–2018
Source: Cancers (Basel). 2023 Jan 31;15(3):870. doi: 10.3390/cancers15030870 (PMC9913805; doi:10.3390/cancers15030870)
Supplement: Supplementary file 1 [file cancers-15-00870-s001.zip › cancers-2140604-supplementary.pdf]

Supplementary files

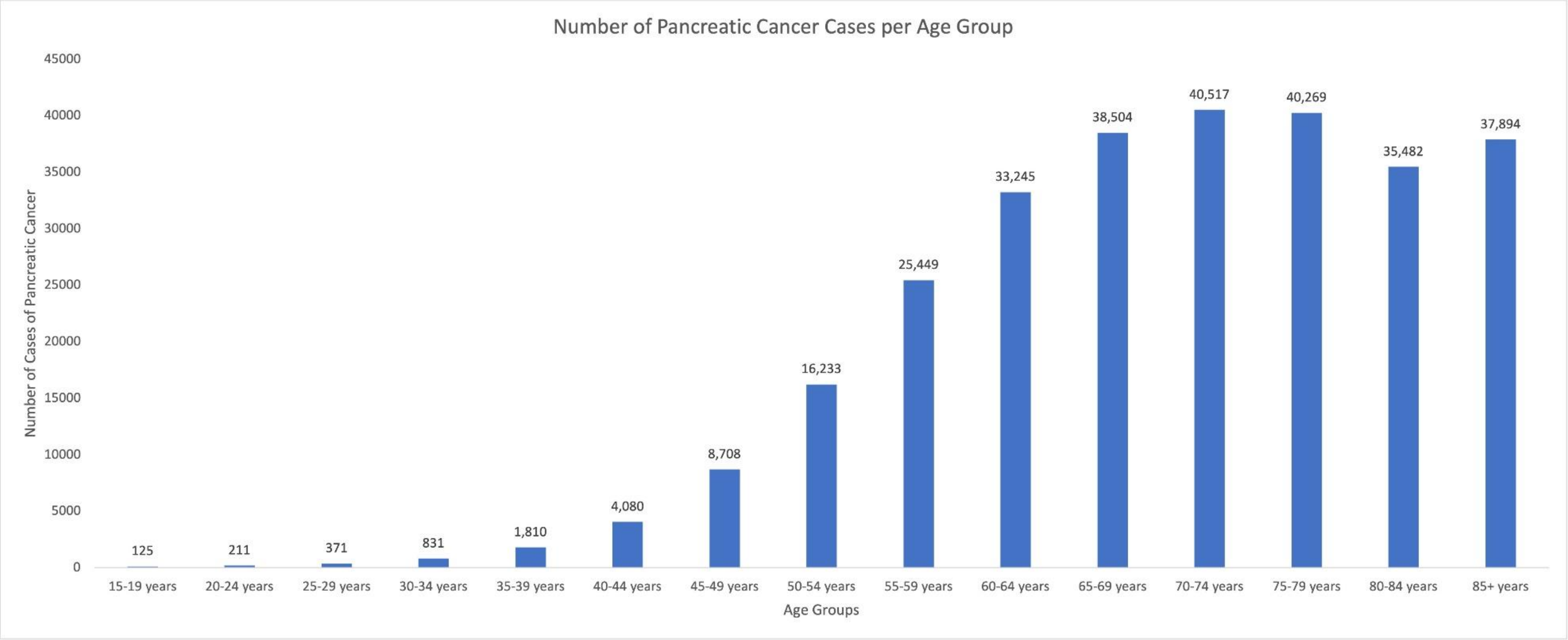

Figure S1: Number of Pancreatic Cancer Cases During the Study Period (2000–2018) by Age Groups.

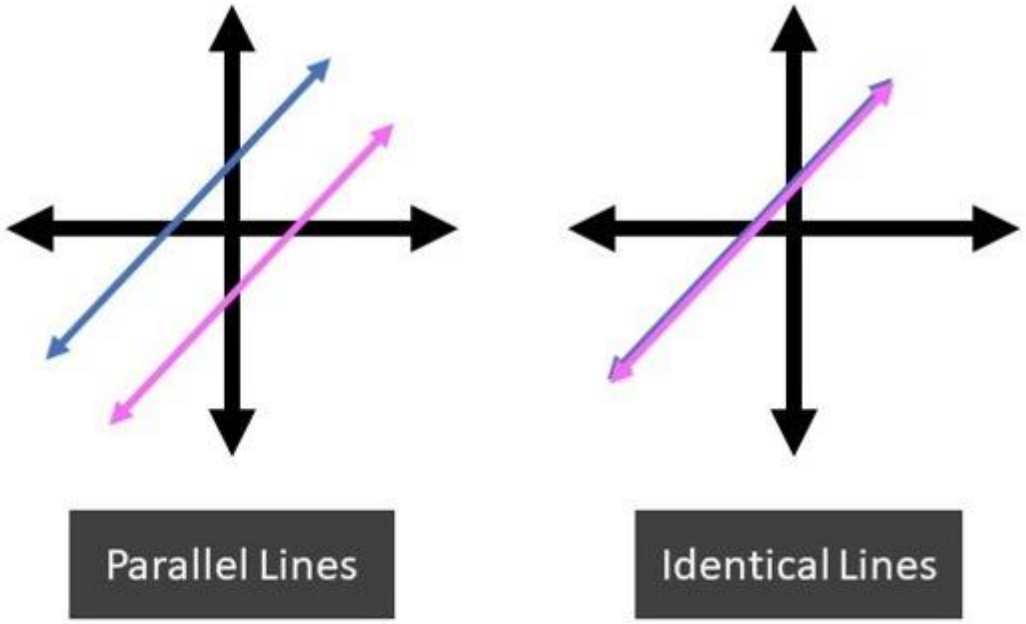

Figure S2: Visual illustration of the test of Parallelism (Parallel Lines) and test of Coincidence (Identical Lines).
